# Quantitative assessment of LASSO probe assembly and long-read multiplexed cloning

**DOI:** 10.1186/s12896-019-0547-1

**Published:** 2019-07-24

**Authors:** Syukri Shukor, Alfred Tamayo, Lorenzo Tosi, H. Benjamin Larman, Biju Parekkadan

**Affiliations:** 1 Center for Surgery, Innovation, and Bioengineering, Department of Surgery, Massachusetts General Hospital, Harvard Medical School and the Shriners Hospitals for Children, 51 Blossom Street, Boston, MA 02114 USA; 2 Division of Immunology, Department of Pathology, Johns Hopkins University, Baltimore, MD USA; 3 Harvard Stem Cell Institute, Cambridge, MA 02138 USA; 4 Department of Biomedical Engineering, Rutgers University, Piscataway, NJ 08854 USA

**Keywords:** Long adapter single-stranded oligonucleotides (LASSO), Genomic libraries, Multiplex PCR, Multiplex cloning

## Abstract

**Background:**

Long Adapter Single-Stranded Oligonucleotide (LASSO) probes were developed as a novel tool for massively parallel cloning of kilobase-long genomic DNA sequences. LASSO dramatically improves the capture length limit of current DNA padlock probe technology from approximately 150 bps to several kbps. High-throughput LASSO capture involves the parallel assembly of thousands of probes. However, malformed probes are indiscernible from properly formed probes using gel electrophoretic techniques. Therefore, we used next-generation sequencing (NGS) to assess the efficiency of LASSO probe assembly and how it relates to the nature of DNA capture and amplification. Additionally, we introduce a simplified single target LASSO protocol using classic molecular biology techniques for qualitative and quantitative assessment of probe specificity.

**Results:**

A LASSO probe library targeting 3164 unique *E. coli* ORFs was assembled using two different probe assembly reaction conditions with a 40-fold difference in DNA concentration. Unique probe sequences are located within the first 50 bps of the 5′ and 3′ ends, therefore we used paired-end NGS to assess probe library quality. Properly mapped read pairs, representing correctly formed probes, accounted for 10.81 and 0.65% of total reads, corresponding to ~ 80% and ~ 20% coverage of the total probe library for the lower and higher DNA concentration conditions, respectively. Subsequently, we used single-end NGS to correlate probe assembly efficiency and capture quality. Significant enrichment of LASSO targets over non-targets was only observed for captures done using probes assembled with a lower DNA concentration. Additionally, semi-quantitative polyacrylamide gel electrophoresis revealed a ~ 10-fold signal-to-noise ratio of LASSO capture in a simplified system.

**Conclusions:**

These results suggest that LASSO probe coverage for target sequences is more predictive of successful capture than probe assembly depth-enrichment. Concomitantly, these results demonstrate that DNA concentration at a critical step in the probe assembly reaction significantly impacts probe formation. Additionally, we show that a simplified LASSO capture protocol coupled to PAGE (polyacrylamide gel electrophoresis) is highly specific and more amenable to small-scale LASSO approaches, such as screening novel probes and templates.

**Electronic supplementary material:**

The online version of this article (10.1186/s12896-019-0547-1) contains supplementary material, which is available to authorized users.

## Background

Techniques that enable multiplexed amplification of specific DNA sequences from large and complex templates, namely genomes and transcriptomes, are invaluable tools for identifying mutations and functional genomic studies [1, 2]. PCR (polymerase chain reaction) amplification is the most common technique used for exponential enrichment of specific sequences from complex templates because it offers operational simplicity and a measure of specificity. Though more technically challenging, DNA padlock probe techniques are an alternative method for sequence amplification that offers high specificity in multiplex approaches by using two template complementary sequences on the same probe, commonly known as molecular inversion probes (MIPs). However, a major constraint of MIPs is the inability to capture sequences greater than 150 base pairs [3, 4]. In an effort to improve upon padlock probe technology, we have previously shown that Long Adapter Single-Stranded Oligonucleotide (LASSO) probes improve the target capture size limitation of MIPs, and demonstrated the massively multiplexed capture-by-circularization of kilobase long genomic regions (up to ~ 4 kb) from *E. coli* genomic DNA [5].

A critical element of LASSO is the creation of thousands of LASSO probes in a single reaction. Since the sequence identities of LASSO probes cannot be discerned by standard molecular biology techniques such as electrophoretic mobility, we did not have a clear understanding of LASSO probe assembly efficiency and how that relates to successful captures downstream. To this end, we set out to quantify LASSO probe assembly using next-generation sequencing techniques (NGS), and correlate those results to the success rate of LASSO capture. Our results confirm the critical nature of LASSO probe assembly and suggest how this step might be improved.

## Results

### Quality assessment of LASSO probe assembly by NGS

**Fig. 1:**
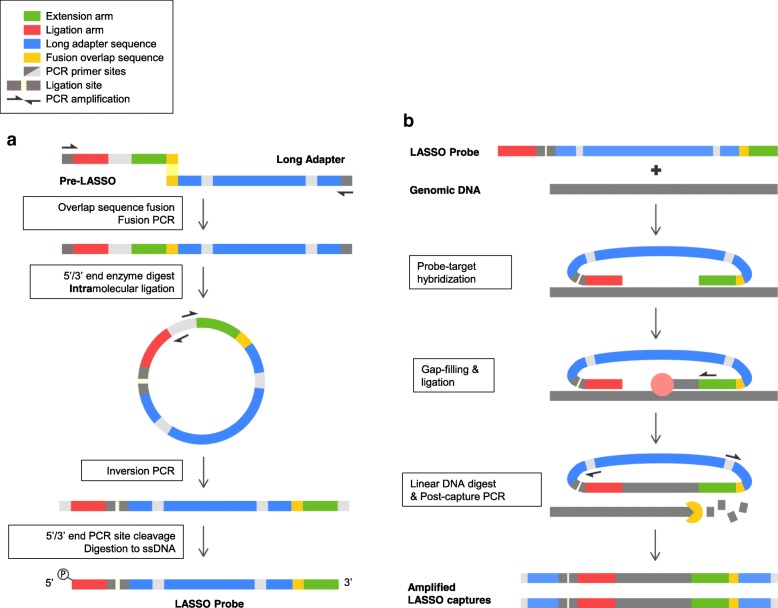
LASSO probe de novo synthesis and capture. **a** Schematic of LASSO probe assembly protocol outline. **b** LASSO target capture outlines probe-template hybridization, target capture and dissociation in a single reaction followed by post-capture PCR

As previously described, a general schematic of LASSO probe assembly and capture is shown in Fig. 1a, b [5]. LASSO probes consist of a constant non-specific linker sequence (long-adapter) flanked by two ~ 50 bps sequences (probe arms) complementary to unique genomic targets. Massively parallel cloning by LASSO requires a multiplexed probe assembly process wherein pre-LASSO probes are fused to adapter sequences and amplified by a PCR-based approach (Fig. 1a). Since LASSO probes created this way are of a uniform size, electrophoretic mobility cannot fully assess the quality of probe assembly. To gain a better understanding of LASSO probe assembly, we focused our analysis on the unique probe arms by using paired-end next-generation sequencing (NGS). This approach allowed us to analyze the 5′ and 3′ sequences of individual LASSO probes within a large library.

In this study, we synthesized a LASSO probe library targeting 3164 unique *E. coli* ORF (open reading frame)-eome sequence as previously described. For comparison, we used two different reaction volumes during a critical step in the probe assembly process, decreasing the reaction volume to 50 μl from the previously used 2 ml [5], thereby dramatically increasing the concentration of DNA in this step. The resulting LASSO probes amplified by PCR were then prepared and submitted for paired-end NGS (2 × 75 bp Illumina MiSeq). Only read pairs from PCR amplicons corresponding to full-length LASSO probes (377 base pairs) were considered in our analysis, and therefore mapped to the probe library. Total raw sequencing reads for 50 μl and 2 ml were 572044 and 457694 reads respectively. Out of the total reads, 300332 (52.5%) and 297049 (64.9%) reads from 50 μl and 2 ml passed pairing and read filtering (Additional file 2: Table S1). Median read depths for all mapped pairs were 69 and 65 for 50 μl and 2 ml, respectively.

Our analysis sheds light on probe arm pair distribution, probe enrichment depth, and probe library coverage. For the purpose of our analysis, a properly assembly LASSO probe is a concordant probe. Concordant probes, as we define them, have read pairs mapped to both 5′ and 3′ ends of one unique reference sequence, confirming that both probe arms originated from the same sequence as intended. However, if read pairs contain probe arms that originate from different probes, then the probe in question has mismatched probe arms and is termed a discordant probe.

**Fig. 2:**
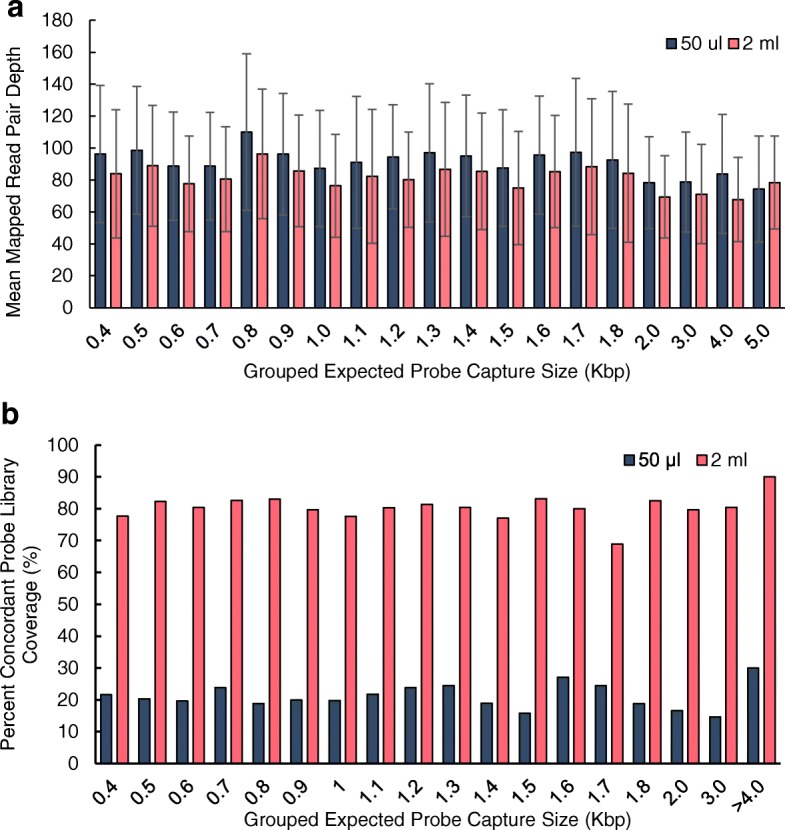
Probe assembly NGS data analysis. **a** Mean read depth of all sequencing reads mapped to the LASSO probe library. The reference probe library sequences (*N* = 3164) were grouped according to ranges of expected capture size in increasing order to highlight biases in probe formation and predict downstream capture performance. Read depth is defined as the number of reads that map to a specific reference sequence. **b** On the horizontal axis, probe library sequences were grouped according to expected probe capture size ranges. The percentages of ORFs represented by concordant probes within these expected capture size ranges were plotted for both LASSO assembly methods. Concordant probes are properly formed probes with paired-end reads that map to a unique probe reference sequence

Concordant probes only accounted for 10.8 and 0.61% of read pairs when we compared probe assembly for the 2 ml and 50 μl and reaction conditions, respectively (Additional file 2: Table S1). This result suggested a very low rate of proper LASSO probe assembly because most probes were discordant. The distribution of LASSO probe enrichment depth, concordant or discordant, was not significantly different between the probe assembly sets and appeared random with regard to probe arm identity, indicating no biases in probe sequencing depth when probes were arranged according to expected target capture length (Fig. 2a). Despite full representation of the probe library in all mapped reads, we observed a dramatic difference in overall concordantly mapped probe coverage of 80.32% (2 ml) versus 20.73% (50 μl). As expected, we also did not detect a relationship between probe library coverage and expected target capture length, indicating that these results are not explained by a sequence bias between assembly protocols (Fig. 2b). These experiments suggest that while concordant probe assembly was highly inefficient in both cases, the 2 ml self-circularization reaction condition resulted in significantly higher library coverage for concordant probes. We next sought to determine if these differences in probe library quality would affect downstream LASSO capture.

### Effect of LASSO probe assembly on multiplexed capture

The LASSO capture and amplification protocol was previously established and is summarized in Fig. 1b [5]. In short, the extension and ligation probe arms hybridize to the start and end of genomic sequence targets. DNA polymerase and template-dependent ligase then fill in the genomic gap between both arms to produce circular LASSO probes containing captured genomic target sequences, which are then amplified during post-capture PCR with a primer set nested within the LASSO probe backbone.

To understand how LASSO capture is affected by the quality of probe assembly, we used NGS to assess multiplexed LASSO capture of an *E. coli* ORFeome, as previously described by Tosi et al. [5]. Additional file 1: Figure S1 plots capture targets ranging from 0.4 to 4.6 kb in size in increasing order, and approximately half of target ORFs are between 0.4 and 1 kb long. Resultant LASSO post-capture PCR products were sheared and sequenced using the Illumina HiSeq platform (50 bp reads). 6982080 and 6968823 total reads were sequenced for captures using the 50 μl or 2 ml probe assembly reaction mixture, respectively (Additional file 3: Table S2). These reads were then filtered for read duplicates and aligned to both *E. coli* gDNA and LASSO backbone sequence commonly shared across all probes to compare levels between genomic capture and probe backbone sequences amplified during post-capture PCR.

**Fig. 3:**
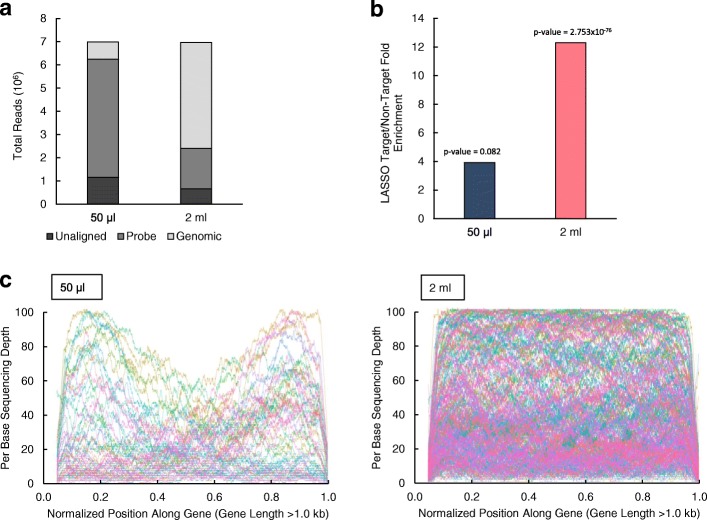
Multiplexed LASSO capture NGS data analysis **a** Distribution of reads aligned to *E. coli* genome and LASSO probe backbone reference sequences. Unaligned reads were also included to reflect total raw sequencing data. **b** Target enrichment values were derived from the ratio of median normalized read depths of targets over median normalized read depths of non-targets. Statistical significance of target enrichment over non-targets was determined using Student’s *t-test* with a 0.99 confidence interval (*p*-value < 0.01. **c** The lengths of LASSO capture targets longer than 1.0 kb with full sequencing coverage were normalized to 1 (Base-pair position normalization). The sequenced depth for each base in captures was then plotted for each normalized position along gene to visualize for target enrichment levels and capture efficiency

Using probes created by the 50 μl assembly reaction condition, 734097 (10.51%) of LASSO capture reads were aligned to the genome, with 5085185 (72.8%) of reads aligning to the LASSO probe linker sequence (Fig. 3a and Additional file 3: Table S2). Somewhat inversely, when probes were created using the 2 ml reaction condition, 4556686 (65.4%) of reads aligned to the *E. coli* genome, while 1743643 (25.02%) of reads aligned to the LASSO linker sequence (Fig. 3a and Additional file 3: Table S2). We next asked if the genome aligned reads were specific capture targets using an analysis analogous with that of a previous report [5]. Targets are specific ORFs targeted by LASSO probes, while non-targets consist of ORFs unrepresented within the probe library and intronic regions. For all reads aligned to *E. coli* gDNA, median normalized read depths for targets/non-targets were 148.9/38.0 for captures performed with probes assembled using the 50 μl reaction condition, and 124.6/10.1 for those using the 2 ml reaction condition, indicating a 4-fold difference in target enrichment in favor of the 2 ml probe assembly reaction condition (Fig. 3b). Statistically significant enrichment of LASSO targets over non-targets was only observed for 2 ml (Student’s *t*-test *p*-value of 2.753 × 10^− 76^ and 0.082 for 50 μl and 2 ml reaction conditions respectively) (Fig. 3b and Additional file 3: Table S2). Using an analysis previously developed [5], we observed similar results on a per base enrichment plot of capture products longer than 1 kb in both captures done with 50 μl and 2 ml ligated probes (Fig. 3c).

While these results demonstrate differences in capture depth, we next asked if capture coverage was affected. Comparing high-quality captures of 50 μl to 2 ml ligated probes at a strict cutoff of ten-fold read coverage, Table 1 shows a two-fold higher LASSO target capture enrichment (590 versus 1282) when using a 2 ml probe assembly reaction condition, while a similar analysis revealed slightly less capture enrichment of non-targets (454 versus 388 non-targets. Plotting Table 1 according to groups of increasing capture target size, we observed that the frequency of high-quality captures for 50 μl to 2 ml ligated probes decreased from 0.4 kb to 1.4 kb and 2.0 kb, respectively (Additional file 1: Figure S1). These results suggest that the probe assembly conditions significantly impact the quality of LASSO capture, and that reducing the concentration of DNA in the probe self-circularization reaction significantly improves probe library quality, which in turn improves downstream capture depth and enrichment.

**Table 1:** High-quality LASSO captures for both target and non-target sequences

	Captured Sequences (> 10-fold Coverage)		Expected Sequences
	50 μl	2 ml	
Targets	590	1282	3164
Non-Targets	454	388	4434

Fold-coverage refers to the number of times that a certain sequence is fully sequenced. High-quality captures were strictly defined as sequences with more than ten-fold sequencing coverage, calculated as a function of sequencing read length (50 bp for HiSeq sequencing platform) multiplied by aligned read depth divided by sequence length

### Simplified single target LASSO capture assessment by gel electrophoresis

Foreknowing the future importance of LASSO capture optimization for new libraries, we further developed a simplified single target LASSO protocol which can be used for small scale cloning or optimization experiments. While LASSO capture quality and efficiency is best evaluated using NGS approaches, initial efforts and optimization are more likely to be performed using inexpensive standard molecular biology techniques, such as gel electrophoresis. To better understand the limitations of this approach, we employed high-resolution polyacrylamide gel electrophoresis (PAGE) and highly sensitive dyes to quantify LASSO capture of individual targets under simplified conditions. LASSO capture parameters and genomic target similar to that used previously were implemented with some modifications [5]. LASSO probes targeting a single 137 base pair target on the M13 bacteriophage genome were designed and synthesized as mature LASSO probes to eliminate LASSO assembly related errors and non-specific capture errors possibly associated with highly multiplexed reactions. We also performed successful LASSO captures of up to 4 kb using this approach, however, these results will be reported in a separate as yet unpublished study [6]. Post-capture PCR reactions were then resolved on both 4% agarose and 4–12% polyacrylamide gels.

**Fig. 4:**
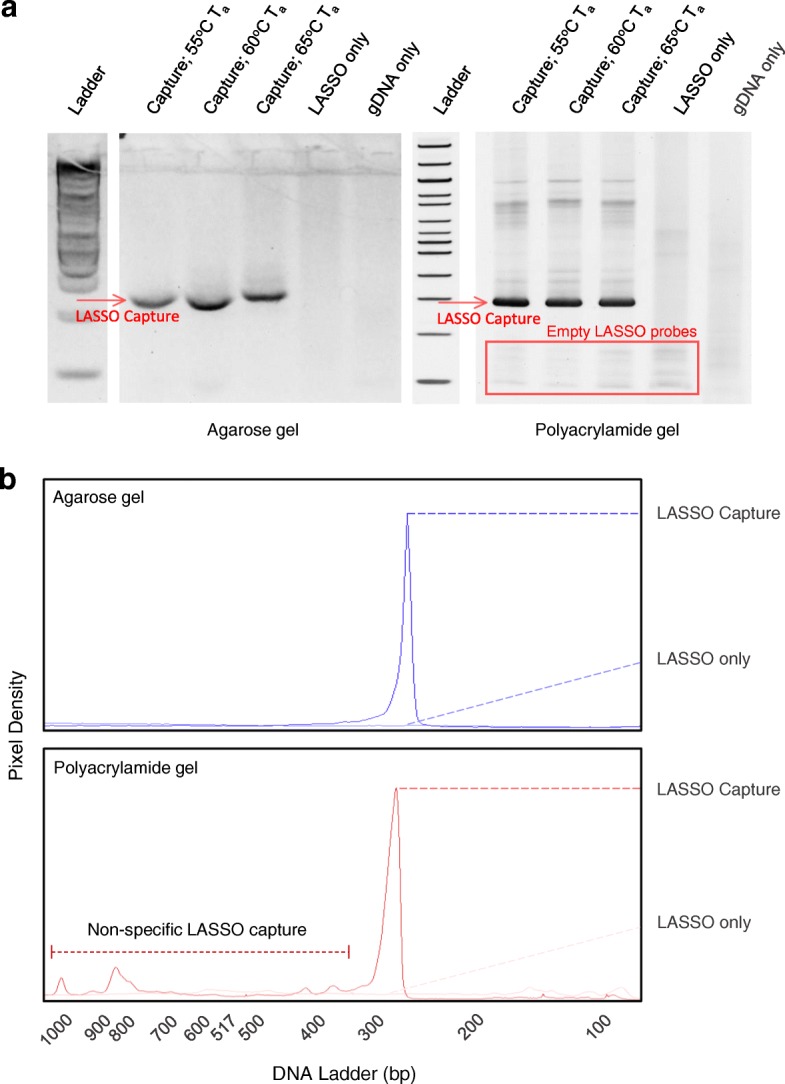
Single-Target LASSO capture gel resolvability. **a** Single target captures resolved on agarose (left) and polyacrylamide gels (right). Both gels are scaled to PAGE DNA ladder, with the major band slightly underneath the 300 bp ladder band. For control lanes, GDNA only control only contains M13 genomic DNA while LASSO only controls only contain M13_137 probes. Annealing temperatures for captures are denoted by “T_a_”, while gDNA and LASSO probe only controls were done at 60 °C **b** Pixel density plots of single target LASSO captures on agarose and PAGE were obtained by ImageJ plot lane functions. In both plots, LASSO captures (60 °C T_a_) are overlaid with a LASSO probe only control to compare non-specific capture with baseline pixel density

A major band of 267 base pairs in size, consisting of 137 bp of capture target and two ~ 50 bp LASSO probe linker sequences, was resolved on agarose gels and PAGE (Fig. 4a). However, PAGE but not agarose gels resolved a series of minor bands ranging from < 0.1 to 0.2 kb, which are likely unreacted LASSO probes. A minor band laddering was also resolved by PAGE but not agarose throughout all capture lanes upwards of 0.3 to 1 kb. These bands were absent in all control lanes, suggesting non-specific LASSO capture or multimer formation during PCR amplification. Pixel density plots from imaged PAGE gels, assigning bands above 0.3 kb as noise, estimate the capture-to-noise ratio to be ~ 10 using this technique under simplified capture conditions (Fig. 4b and Additional file 4: Table S3). These results suggest that PAGE coupled to high sensitivity dyes should be employed instead of agarose gels during LASSO optimization, and that the highest observable enrichment using this simplified system is 10-fold over background.

## Discussion

We envision an array of future applications for LASSO, such as exploring long non-coding RNAs, producing chromosome-scale functional gene fragments, and preparing long-read sequencing libraries [7]. In order to realize the value of LASSO for these diverse applications, optimizing this powerful cloning tool for robustness is critical. This study introduced quantitative and highly sensitive quality control steps to the established LASSO probe assembly and target capture protocol. Here, we used NGS approaches to analyze LASSO probe assembly and correlated our results with a previously established capture and cloning NGS analytical approach.

**Fig. 5:**
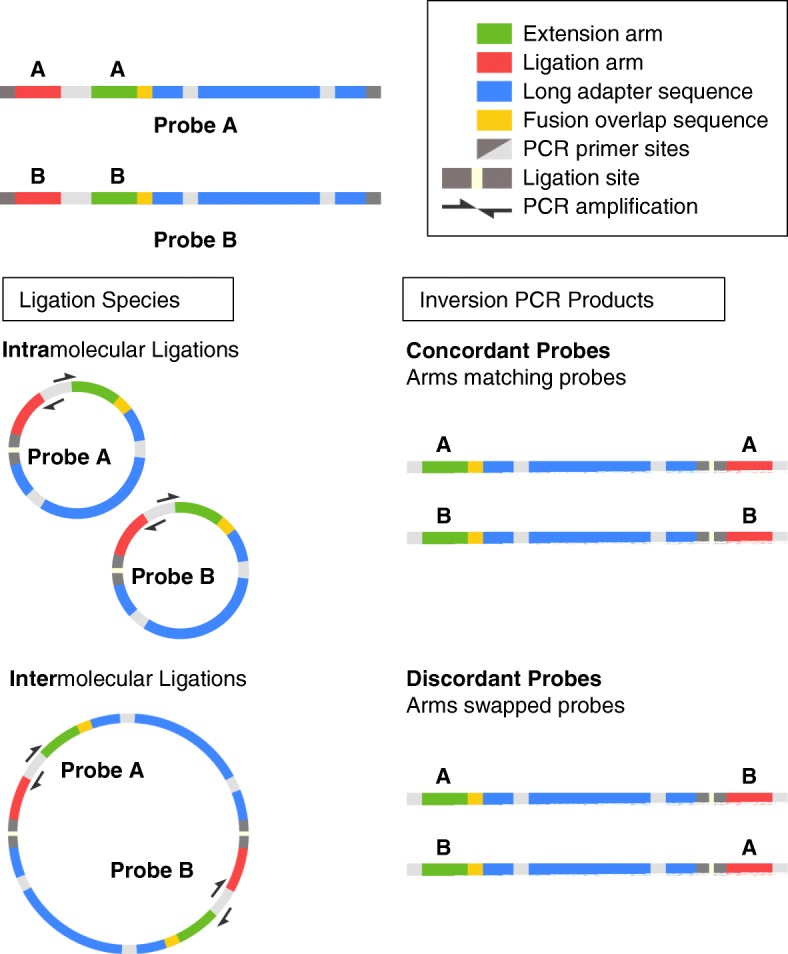
Possible ligation events within probe assembly. Both Probe A and Probe B are formed after the Fusion PCR step prior to restriction enzyme digest and self-circularization. Examples of intermolecular ligation errors formed during self-circularization step (ligation reaction) of the LASSO probe assembly. Resultant discordant probes from intermolecular ligations shown here have mismatched arms from separate probe sequences

A critical phase in the LASSO probe assembly protocol is the self-circularization step (Fig. 1a), wherein the commonly shared Eco-RI digested probe ends are intramolecularly ligated to each other. When attempting to generate thousands of probes in a single reaction by this manner, there exists a strong possibility that intermolecular ligations would manifest as mismatched probe arms on a mature LASSO probe (Fig. 5). Indeed, our analysis revealed that most LASSO probes assembled this way were mismatched (Additional file 2: Table S1).

From the outset, we reasoned that modulating the probe assembly reaction conditions at the critical step of self-circularization would impact LASSO probe assembly and possibly, in turn, capture. The two conditions we compared were not significantly different in the efficiency of probe assembly, given that more than 90 % of LASSO probes contained mismatched arms in either case. However, using a probe assembly protocol that decreases the DNA concentration during ligation by ~ 40 fold dramatically improved LASSO probe library coverage to ~ 80% (Fig. 2 and Additional file 2: Table S1) [5]. Importantly, only LASSO probes generated with our 2 ml self-circularization assembly protocol with ~ 80% probe library coverage produced significant successful captures (Fig. 3), suggesting that library coverage is critical for successful LASSO capture rather than concordant probe depth-enrichment. These experiments confirm that the self-circularization step is a critical area for further improvements to the LASSO protocol. In addition, we introduce an abbreviated LASSO protocol that bypasses de novo probe assembly altogether with limited scalability (Fig. 4), but whereby nearly all probes are properly assembled; providing a feasible standard upon which to base future probe assembly optimization efforts.

## Conclusions

Our results demonstrate that self-circularization DNA concentration significantly impacts probe formation. NGS analysis suggests that the uniformity of concordant LASSO probe coverage is critical for efficient target capture. In addition, we introduce a single target LASSO capture protocol which does not require de novo LASSO probe assembly, making the technique more accessible to inexperienced users and more efficient for small scale approaches, such as screening novel LASSO probes or templates.

## Methods

### LASSO probe component design

#### Pre-LASSO probes

As previously reported [5], our Pre-LASSO design algorithm parsed 4140 cDNA sequences of *E. coli* K-12 MG1655 (Ensemble) to produce a pool of pre-LASSO probes targeting 3164 ORFs. Resulting Pre-LASSO probe sequences were synthesized as pooled single-stranded DNA oligonucleotides derived from programmable DNA microarray (Custom Array Inc.) approximately 160 bp long and had the design as 5′-GAGTATTACCGCGGCGAATTC-ligation arm (variable)-AACACTTCTTGCGGCGATGGTTCCTGGCTCTTCGATC-extension arm (variable)-AGAGAAGTCCTAGCACGGTAACC-3′. The pre-LASSO algorithm only targeted *E. coli* ORFs larger than 400 bp and eliminated extension and ligation arm sequences containing EcoRI digestion sites.

### Long adapter sequence

Table 2 shows the 242 bp Long Adapter sequence used to assemble LASSO probes for *E. coli* ORFeome captures. While the M13_137 sequence was fully synthesized LASSO probes for single target M13 Bacteriophage genomic capture. The long adaptor has a 5′-AGAGAAGTCCTAGCACGGTAACC sequence similar to the 3′ end of pre-LASSO probes functioning as the overlap sequence during Fusion PCR (Fig. 1a).

**Table 2:** LASSO long adapter sequence

Sequence Name	Sequence (5′-3′)
Long Adapter for Multiplexed LASSO Probes	AGAGAAGTCCTAGCACGGTAACCTCCGAGGATGTCATCAAAGAGTTTAAAGAGTTTATGAGATTTAAGGTCAAGATGGAGGGAAGCGTCAACGGACACGAGTTCGAGATTGAGGGAGAAGGAGAAGGCCGGCCTTACGAGGGCACACAAACCGCTAAGCTCAAGGTCACAAAAGGAGGACTAACTATAACGGTCCTAAGGTAGCGAACCCTCCCCTTCTCCTGGGATATTCTGAGCCCTCAGTTCCAGTACGGAAGCGAATTCCAGCTT

### LASSO probe assembly

The LASSO probe assembly protocol is the same as that previously described [5], with exceptions during the ligation protocol (self-circularization). The primers used during Fusion PCR were FusionBlaF and RFP200EcoR1 (Table 3). Approximately 45 μl of solution containing gel-purified fusion PCR product as described were digested by adding 5 μl of CutSmart 10X buffer (NEB) and 1 μl (20 units/μl) of EcoRI-HF (NEB) for 1 h at 37 °C followed by a denaturation step for 10 min at 80 °C. EcoRI digested DNA was purified with Agencourt AMPure XP beads (Beckman Coulter) and eluted in 40 μl ddH_2_O.

**Table 3:** Primer sequences for LASSO assembly and capture

Primer	Sequence (5′-3′)
BlaF	GAGTATTACCGCGGCGAATTC
RFPR200EcoRI	AAGCTGGAATTCGCTTCCGTACTGGAACTGAGGGC
SapINew	GGTTCCTGGCTCTTCGATC
TiolNew	A*T*C*GCCGCAAGAAGTGTU
PCR1kbCaptF400	GTGAAACTCAGAGGAACCAACTTCC
ICeul200CaptF	CTCCCCTTCTCCTGGGATATTCTG
M13_137_R(Tiol)	TTCAAAGCGAACCAGACCGG
M13_137_F(SapINew)	/5Phos/GCAATCCGCTTTGCTTCTGAC

“*” symbol denotes phosphorothioate bonds while /5Phos/ indicates a phosphorylated 5′ end

#### 50 μl ligation protocol

For the 50 μl self-circularization method, the reaction was performed in a total volume of 50 μl 1X T4 Ligase Buffer (NEB) containing approximately 5 ng EcoRI-digested fusion PCR product (0.1 ng/μl) and 1 μl of T4 DNA ligase (400 units) which was lastly added into the reaction. The reaction was performed in a thermocycler (Eppendorf Mastercycler) for 1 h at 25 °C followed by a 10-min denaturation step at 65 °C.

#### 2 ml ligation protocol

As for the 2 ml ligation protocol, self-circularization reaction was performed in a 15 ml conical tube (Corning). A reaction volume of 2 ml 1X T4 Ligase Buffer (NEB) containing approximately 5 ng of EcoRI digested fusion PCR product (0.1 ng/μl). Ten microliters of T4 DNA ligase (4000 units) was the final component added into the reaction. Ligation was performed in a 16 °C water bath for 16 h. The reaction was then vacuum spun for 3 h in a Savant SpeedVac concentrator (Thermo Scientific). Concentrated ligation was adjusted to 100 μl with ddH_2_O, purified by Agencourt AMPure XP beads (Beckman Coulter), and finally eluted in 50 μl ddH_2_O.

For ligations from 50 μl and 2 ml methods, 1 μl of λ-Exonuclease (5 U/μl) (NEB) and 1 μl of Exonuclease I (20 U/μl) (NEB) was added directly into the PCR tubes containing self-circularized DNA to digest any linear DNA. Digestion parameter was 30 min at 37 °C followed by 20 min at 80 °C.

Inversion PCR was performed in a 25 μl reaction. Ten microliters of circularized DNA from 50 μl or 2 ml ligation methods was aliquoted with 2.5 μl of 10X Klentaq Mutant Buffer (DNA Polymerase Technology), 0.5 μl of deoxynucleotides (dNTPs) (NEB), 10 μl ddH_2_O, 1 μl of primers TiolNew and SapINew, and finally 0.1 μl of Omni-Klentaq LA (DNA Polymerase Technology). Both SapI and TiolNew primers anneal with opposite orientations on the conserved sequence connecting the ligation and extension arms of pre-LASSO sequences (5′-AACACTTCTTGCGGCGATGGTTCCTGGCTCTTCGATC-3′). The SapINew primer contains a SapI/BspQI restriction site. TiolNew primer has 3 phosphorothioate bonds on its 5′-end, indicated by “*” in between primer bases. On the 3′ end of TiolNew, a “U” indicates a deoxy-uracil moiety included for USER enzyme (NEB) cleavage (Table 3). The PCR thermal profile was as follows: - 5 min at 95 °C; 30 cycles of 15 s at 95 °C, 20 s at 55 °C, and 40 s at 72 °C; ending with an elongation step for 5 min at 72 °C.

The inversion PCR products were subsequently purified by Agencourt AMPure XP beads (Beckman Coulter) and finally eluted in 40 μl ddH_2_O. The concentration of purified inversion PCR products was determined by Nanodrop. Twenty microliters of purified inversion PCR products were then brought up to 40 μl with 4 μl 10X CutSmart Buffer (NEB) and 16 μl of ddH_2_O. The following enzymes were sequentially added (thermal profiles included): i) 1 μl of BspQI restriction enzyme (NEB) (1 h at 50 °C; 20 min at 80 °C), ii) 1 μl of Lambda Exonuclease (NEB) (30 min at 37 °C; 20 min at 80 °C), and iii) 2 μl USER (Uracil-Specific Excision Reagent) enzyme (NEB) (30 min at 37 °C).

The sequential enzyme digests above i) cleave SapINew primer site, ii) digest DNA strands without 5′ phosphorothioate bonds, and iii) cleave TioINew primer site to yield capture-ready mature LASSO probes.

### Paired-end NGS for inversion PCR products

Non-sheared Inversion PCR products were sent for paired-end NGS as per manufacturer’s protocol (2 × 75 bp MiSeq Reagent Nano Kit V3) (Illumina). Raw paired-end sequencing outputs (R1 and R2 files) from Illumina MiSeq were processed and analyzed using a computational pipeline to quantitate distribution of properly formed (concordant) probes over improperly formed (discordant) probes depending on how read pairs align to the probe library reference sequences (*N* = 3164). BBTools Repair parsed reads from R1 and R2 files and fixed read pairs into the correct order based on read identifiers assigned by the Illumina sequencer. Reads in one file without a pair in another file (singletons) were discarded [8]. Trimmomatic default parameters were then used to trim Illumina adapter sequences from paired read ends. TruSeq3 adapter sequences were used as reference for adapter trimming [9]. Read pairs were mapped to a probe library consisting of 3164 unique LASSO probe sequences that are 377 bp long by Bowtie2 (‘--very-sensitive’ preset) [10]. Post alignment, SAMtools selected only for probes with MAPQ alignment scores equals to or more than 30. These alignments are likely to be correctly mapped with a probability of 0.999. Reads were then ordered and indexed before sequencing duplicate removal by Picard (REMOVE_SEQUENCING_DUPLICATES = true). Our LASSO capture computational workflow includes a post-alignment duplicate removal step to account for the drawbacks of NGS sequencing platforms [11, 12]. Further SAMtools filtering steps separated mapped read pairs as concordant or discordant probes [13]. All mapped reads were grouped according to expected probe capture sizes in increasing order. Standard deviation read depths for probes in each group were then obtained (Excel).

### Multiplexed kilobase-long targets LASSO captures

Overall, multiplexed LASSO ORF-eome captures were performed as previously described by Tosi et al. [5]. Both gap-filling and linear DNA digestion solution were prepared fresh for every capture experiment. Components of the gap-filling solution were 5 μl 10X Ampligase DNA buffer (Epicentre), 2 μl dNTP solution (1 mM), 1 μl Ampligase DNA Ligase (Epicentre), 0.2 μl Omni Klentaq LA, and 42 μl ddH_2_O. Linear DNA digest solution components were 8 μl Exonuclease I enzyme (NEB), 8 μl Exonuclease III enzyme (NEB), 8 μl EcoRI enzyme and 8 μl ddH_2_O.

One microliter of capture-ready LASSO probes (~ 10 ng), 2 μl of *E. coli* K-12 (Migula) gDNA (500 ng), 1.5 μl 10X Ampligase Buffer, and 10.5 μl ddH_2_O for a total reaction volume of 15 μl. Probe-genomic target hybridization was done for 5 min at 95 °C followed by 60 min at 60 °C. Five microliters of gap-filling mix was subsequently added into the reaction and incubated for 30 min at 60 °C. After an incubation step for 3 min at 95 °C to dissociate probes from targets, 2 μl of linear DNA digestion solution was added with an incubation time of 1 h at 37 °C followed by a final denaturation step for 20 min at 80 °C.

Post-Capture PCR reaction consisted of 5 μl of LASSO captures, 2.5 μl 10X Klentaq Mutant Buffer (DNA Polymerase Technology), 0.5 μl dNTP solution (10 mM), 1 μl of each primers IceuI200CaptF200 and PCR1kbCaptF400, 15 μl of ddH_2_O, and 0.1 μl Omni-Klentaq LA DNA polymerase (Table 3). PCR thermal profile were: 5 min at 95 °C; 30 cycles of 15 s at 95 °C, 20 s at 55 °C, and 2 min at 72 °C; and a final extension step of 5 min at 72 °C.

### Single-end NGS for multiplexed LASSO captures

LASSO capture sequencing library preparation and computational analysis were performed as previously described with exceptions described below [5]. Trimmomatic was used for Illumina adapter sequence clipping and poor quality read filtering. Two separate Bowtie 2 read alignments were done against *E. coli* K-12 genome and LASSO backbone sequence (extension and ligation arm sequences excluded) [9]. SAMtools filtered only for high quality reads with MAPQ scores of at least 30. Another read filtering step with Picard and SAMtools removed duplicate reads [13].

Filtered alignment files were then analyzed with Bedtools *genomecov* and *coverage* function [14]. Reference *.bed* files were divided as target ORFs, untargeted ORFs, and intergenic sequences to account for the full *E. coli* genome sequence. Bedtools output files contained information on read depth per base pair, sequence fraction coverage, normalized read counts, and sequencing statistical summary, which were analyzed with Excel (Microsoft). Our analysis compared target ORFs and non-targets - untargeted ORFs and intergenic sequences combined. Statistical significance between target and non-target enrichment was performed as previously described [5].

### Single target M13 Bacteriophage LASSO capture & semi-quantitative analysis

#### Probe design

Unlike LASSO probes used in the *E. coli* ORF-eome captures, single-target LASSO probes were synthesized de novo as GeneBlock Fragments (IDTDNA). These probes were designed with a ~ 400 bp Long Adapter (Table 4) flanked with ligation/extension arms targeting a 137 base pair sequence within the M13 Bacteriophage genome, a low complexity single-stranded circular genome. The ligation arm of this probe is the same as previously used while the extension arm was determined by our LASSO design algorithm [5]. The total length for this fully synthesized single target probe is 540 bp without TiolNew and SaplNew primer sites.

**Table 4:** Single-target M13 LASSO probe design

Sequence Name	Sequence (5′-3′)
M13_137	GCAATCCGCTTTGCTTCTGACTATAATAGTCAGGGTAAAGACCTAGAGAAGTCCTAGCACGGTAACCTCCGAGGATGTCATCAAAGAGTTTAAAGAGTTTATGAGATTTAAGGTCAAGATGGAGGGAAGCGTCAACGGACACGAGTTCGAGATTGAGGGAGAAGGAGAAGGCCGGCCTTACGAGGGCACACAAACCGCTAAGCTCAAGGTCACAAAAGGAGGACTAACTATAACGGTCCTAAGGTAGCGAACCCTCCCCTTCTCCTGGGATATTCTGAGCCCTCAGTTCCAGTACGGAAGCAAAGCCTATGTTAAACACCCTGCCGACATCCCTGACTATCTGAAGCTCTCCTTCCCTGAAGGCTTCAAGTGGGAGAGATTCATGAACTTCGAGGACGGAGGCGTGGTGACAGTCACACAAGATAGCACCCTCCAGGACGGAGAGTTTATTTATAAGGTGAAACTCAGAGGAACCAACTTCCCCTCCGATGGCCCTGTCATGAATTCTTGGAGTTTGCTTCCGGTCTGGTTCGCTTTGAA

#### Mature LASSO probe generation

For preparative purposes, we used primers targeting the ligation and extension arms in the PCR reaction to amplify the full length of LASSO probes. PCR reaction consisted 1 μl of M13_137 probe (10 ng), 5 μl Multiplex PCR 5X Master Mix (NEB), 17 μl ddH_2_O, and 1 μl each of primers M13_137_R and M13_137_F (Table 3). PCR thermal profile was 2 min at 95 °C; 30 cycles of 20 s at 95 °C, 35 s at 55 °C, and 1 min at 68 °C; with 5 min at 68 °C. PCR reaction was run in 1.0% agarose gel. Amplified band (540 bp) was excised and purified with QIAEX II Gel Extraction Kit (Qiagen); 10 μl of QXI silica-gel particles, and eluted in 25 μl ddH_2_O. The gel extraction yields were quantitated with Nanodrop.

Mature probes were produced via digestion of gel extracted PCR products into single-stranded DNA by Lambda Exonuclease. Enzyme activity prefers 5′ phosphorylated end on the DNA strand amplified by the M13_137_F primer. The reactions were done with 13.5 μl of gel extracted LASSO probes, 2.5 μl 10X CutSmart Buffer, 9 μl of ddH_2_O and 1 μl Lambda Exonuclease at 37 °C for 1 h.

#### Single target LASSO captures

Probe-target hybridization reaction was set-up in triplicates of 15 μl reactions in PCR tubes. Each reaction consists 1 μl of M13_137 probes (~ 5.0 fmol), 1 μl of M13mp18 Bacteriophage ssDNA (~ 0.5 fmol) (NEB), 11.5 μl of ddH_2_O, and 1.5 μl of 10X Ampligase Buffer. In contrast to what was previously done, M13_137 captures were done with a ten-fold molar excess of probes to genomic DNA [5]. Hybridization step was initiated with a 5-min denaturation step at 95 °C followed by a hybridization step with hybridization temperatures (T_a_) of 55 °C, 60 °C, and 65 °C for each reaction using our thermocycler’s gradient temperature setting.

Five microliters of gap-filling solution was subsequently added – 3.85 μl ddH_2_O, 0.5 μl Ampligase Buffer, 0.2 μl dNTP solution, 0.25 μl of Ampligase DNA ligase (Epicentre), and 0.2 μl Omni-Klentaq LA DNA Polymerase. Reactions were then incubated according to their respective T_a_ at 55 °C, 60 °C, and 65 °C for 30 min ended by a 3-min denaturation step at 95 °C.

#### Post-capture PCR

Five microliters of single-target LASSO captures was mixed with 5 μl Multiplex PCR 5X Master Mix, 13 μl ddH_2_O, and 1 μl each of LASSO post-capture primers PCR1kbCaptF400 and ICeuI200CaptF (Table 3). PCR thermal profiles were as follows: 3 min at 95 °C; 30 cycles of 20 s at 95 °C, 35 s at 55 °C, and 45 s at 68 °C; with 3 min at 68 °C.

#### LASSO post-capture PCR visualization

A 4.0% agarose gel was cast and pre-stained with ethidium bromide. The whole post-capture PCR reaction was mixed with 5 μl of 6X Purple Gel Loading Dye (NEB). Novex 4–12% 12-well TBE Polyacrylamide Gels (Thermo Fisher) were used to resolve 2.5 μl of PCR amplified LASSO captures in 1X Purple Gel Loading Dye. For capture-to-noise ratio quantitation, four two-fold serial dilutions were made starting with a 1X PCR solution containing 8 μl of PCR product (60^o^ C T_a_), 12 μl of ddH_2_O, and 4 μl Purple Loading Dye. Four two-fold serial dilutions of 1 kb Plus DNA Ladder (Thermo Fisher) were included for relative capture DNA mass estimation. PAGE for DNA quantitation was performed in duplicates. All PAGE gels were stained with SYBR Gold Nucleic Acid Gel Stain (Thermo Fisher) for 10 min before visualization via a gel imager (FastGene).

#### LASSO capture signal-to-noise ratio quantitation

Since captures with varying annealing temperature yielded no significant difference on PAGE, we randomly chose the capture done with 60 °C for signal-to-noise ratio quantitation. Stained PAGE gels were imaged with a gel imager and saved in high resolution .*tiff* format with similar exposure levels. Gel color profiles were inverted – black bands on white background - and analyzed with ImageJ (Fiji). Uniform vertical rectangular selections were done on every gel lane to plot for pixel density of resolved bands. Using ImageJ Plot Lanes function, standard curves correlating pixel density to expected DNA mass were plotted using DNA ladder bands; derived trendlines were used to estimate DNA mass. Signal-to-noise ratio was obtained only from samples in which its estimated DNA mass from band pixel density was within the linear portion of the trendline. Visible minor bands corresponding to non-specific LASSO capture (excluding bands from empty probes) were gated and classified as noise. Pixel density ratios between major bands versus total noise, or minor bands, were derived [13].

## Additional files

Additional file 1: **Figure S1.** LASSO Probe Library Target Expected & Observed Frequency. Histogram groups the frequency of expected and observed high-quality LASSO ORF-eome target captures (More than ten-fold depth coverage) grouped in increasing capture sizes from 0.4 to > 4.0 kb. (PPTX 43 kb)

Additional file 2: **Table S1.** NGS Read Accounting of Sequenced Assembled LASSO Probes The table shows raw sequencing output (MiSeq 2 × 75 bp platform) of inversion PCR products before and after read pairing, sequencing duplicate removal, and alignment using our NGS pipeline. (XLSX 8 kb)

Additional file 3: **Table S2.** NGS Read Accounting of Sequenced LASSO *E. coli* ORF-eome Captures. The table shows raw sequencing output (HiSeq 1 × 50 bp platform) of PCR amplified multiplexed ORF-eome capture. Reads were first aligned to both *E. coli* genome and LASSO probe backbone sequence, then read mapped exclusively to genomic DNA was cleaned, filtered, and separated according to targets or non-targets (untargeted ORFs and intergenic regions). Also included are the median read depths of gDNA sequencing enrichment. (XLSX 9 kb)

Additional file 4: **Table S3.** Single Target Capture ImageJ Pixel Density Plot. Single target LASSO captures were visualized on PAGE gels and imaged. Major and minor bands were highlighted using ImageJ to obtain pixel density plots. (XLSX 8 kb)

## Data Availability

The dataset(s) supporting the conclusions of this article is(are) included within the article (and its additional file(s)). All the custom parameters used in Trimmomatic, Bowtie 2, Picard, Samtools, and Bedtools are provided in GitHub: https://github.com/sykrishukor94/LASSO-NGS-Pipelines

## Abbreviations

LASSO: Long adapter single stranded oligonucleotide
NGS: Next generation sequencing
ORF: Open reading frame
PAGE: Polyacrylamide gel electrophoresis
PCR: Polymerase chain reaction
