# Supplementary figures and images for: Quantitative assessment of LASSO probe assembly and long-read multiplexed cloning
Source: BMC Biotechnol. 2019 Jul 24;19:50. doi: 10.1186/s12896-019-0547-1 (PMC6657055; doi:10.1186/s12896-019-0547-1)

## Slide 1
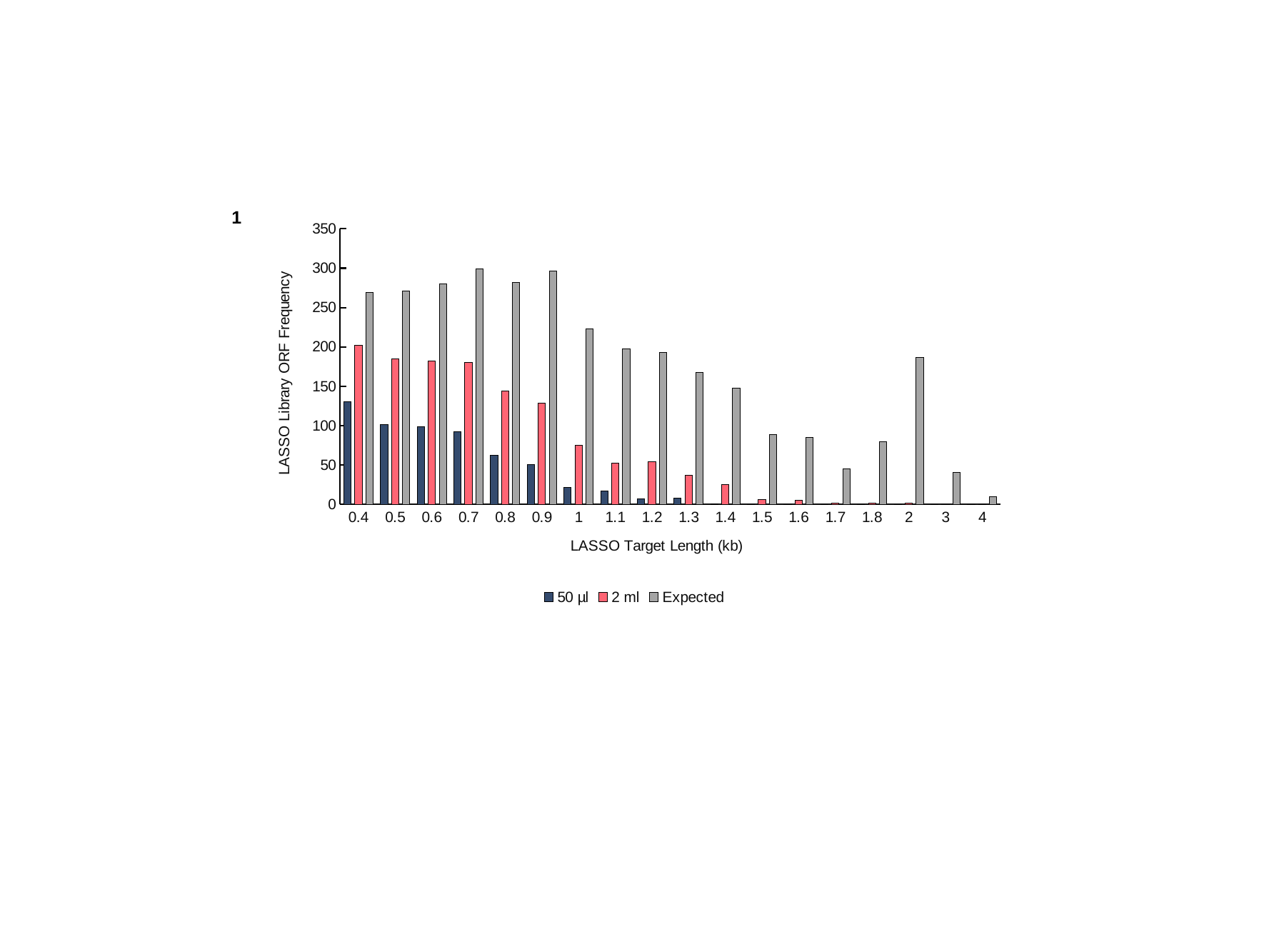

1
### Chart
| Category | 50 µl | 2 ml | Expected |
|---|---|---|---|
| 0.4 | 130.0 | 202.0 | 269.0 |
| 0.5 | 101.0 | 185.0 | 271.0 |
| 0.6 | 99.0 | 182.0 | 280.0 |
| 0.7 | 92.0 | 180.0 | 299.0 |
| 0.8 | 62.0 | 144.0 | 282.0 |
| 0.9 | 51.0 | 129.0 | 296.0 |
| 1 | 22.0 | 75.0 | 223.0 |
| 1.1000000000000001 | 17.0 | 52.0 | 198.0 |
| 1.2 | 7.0 | 54.0 | 193.0 |
| 1.3 | 8.0 | 37.0 | 168.0 |
| 1.4 | 1.0 | 25.0 | 148.0 |
| 1.5 | 0.0 | 6.0 | 89.0 |
| 1.6 | 0.0 | 5.0 | 85.0 |
| 1.7 | 0.0 | 2.0 | 45.0 |
| 1.8 | 0.0 | 2.0 | 80.0 |
| 2 | 0.0 | 2.0 | 187.0 |
| 3 | 0.0 | 0.0 | 41.0 |
| 4 | 0.0 | 0.0 | 10.0 |

Supplement: Supplementary file 1 — Figure S1. LASSO Probe Library Target Expected & Observed Frequency. Histogram groups the frequency of expected and observed high-quality LASSO ORF-eome target captures (More than ten-fold depth coverage) grouped in increasing capture sizes from 0.4 to > 4.0 kb. (PPTX 43 kb) [file 12896_2019_547_MOESM1_ESM.pptx]
